# Endodontic-Like Oral Biofilms as Models for Multispecies Interactions in Endodontic Diseases

**DOI:** 10.3390/microorganisms8050674

**Published:** 2020-05-06

**Authors:** Dejana Lukic, Lamprini Karygianni, Manuela Flury, Thomas Attin, Thomas Thurnheer

**Affiliations:** Clinic of Conservative and Preventive Dentistry, Center of Dental Medicine, University of Zurich, 8032 Zurich, Switzerland; lukicdejana2015@gmail.com (D.L.); lamprini.karygianni@zzm.uzh.ch (L.K.); manuela.flury@zzm.uzh.ch (M.F.); thomas.attin@zzm.uzh.ch (T.A.)

**Keywords:** endodontic pathogens, endodontic-like multispecies biofilm, pulpitis, pulp necrosis, quantitative real-time PCR (qPCR), CLSM, FISH

## Abstract

Oral bacteria possess the ability to form biofilms on solid surfaces. After the penetration of oral bacteria into the pulp, the contact between biofilms and pulp tissue may result in pulpitis, pulp necrosis and/or periapical lesion. Depending on the environmental conditions and the availability of nutrients in the pulp chamber and root canals, mainly Gram-negative anaerobic microorganisms predominate and form the intracanal endodontic biofilm. The objective of the present study was to investigate the role of different substrates on biofilm formation as well as the separate and collective incorporation of six endodontic pathogens, namely *Enterococcus faecalis, Staphylococcus aureus, Prevotella nigrescens, Selenomonas sputigena, Parvimonas micra* and *Treponema denticola* into a nine-species “basic biofilm”. This biofilm was formed in vitro as a standard subgingival biofilm, comprising *Actinomyces oris, Veillonella dispar,* *Fusobacterium nucleatum, Streptococcus anginosus, Streptococcus oralis, Prevotella intermedia, Campylobacter rectus, Porphyromonas gingivalis,* and *Tannerella forsythia.* The resulting endodontic-like biofilms were grown 64 h under the same conditions on hydroxyapatite and dentin discs. After harvesting the endodontic-like biofilms, the bacterial growth was determined using quantitative real-time PCR, were labeled using fluorescence in situ hybridization (FISH) and analyzed by confocal laser scanning microscopy (CLSM). The addition of six endodontic pathogens to the “basic biofilm” induced a decrease in the cell number of the “basic” species. Interestingly, *C. rectus* counts increased in biofilms containing *E. faecalis, S. aureus, P. nigrescens* and *S. sputigena,* respectively, both on hydroxyapatite and on dentin discs, whereas *P. intermedia* counts increased only on dentin discs by addition of *E. faecalis*. The growth of *E. faecalis* on hydroxyapatite discs and of *E. faecalis* and *S. aureus* on dentin discs were significantly higher in the biofilm containing all species than in the “basic biofilm”. Contrarily, the counts of *P. nigrescens*, *S. sputigena* and *P. micra* on hydroxyapatite discs as well as counts of *P. micra* and *T. denticola* on dentin discs decreased in the all-species biofilm. Overall, all bacterial species associated with endodontic infections were successfully incorporated into the standard multispecies biofilm model both on hydroxyapatite and dentin discs. Thus, future investigations on endodontic infections can rely on this newly established endodontic-like multispecies biofilm model.

## 1. Introduction

Most oral bacteria are commensal [1], but depending on host immune response and dysbiotic microbial interactions rather than on specific pathogens [2], they contribute to oral diseases [3]. Like bacterial species in general, oral bacteria possess the ability to form biofilms on solid surfaces in the presence of nutrient-containing fluids [4]. Biofilms were described decades ago as communities of bacterial cells that are embedded in a polymeric matrix that contains polysaccharides, DNA, RNA, proteins, lipids and other components, adhering to various surfaces and showing different phenotypical and biochemical characteristics compared to their planktonic counterparts [5,6,7,8]. Biofilms protect bacterial cells from host defense mechanisms and antibiotics; likewise, they disseminate planktonic bacterial cells that can cause acute disease. Early investigators assumed that the biofilm formation is a survival mechanism of bacteria while seeking an optimal position for gaining nutrients [4]. Meanwhile, studies have shown that bacterial signal molecules and positioning mechanisms predetermine the position and spatial relationships of biofilms. In other words, the biofilm demonstrates a level of differentiation that requires a sophisticated network of cell–cell signals and a high degree of cellular specialization [9,10].

Diseases of the teeth and the tooth supporting structures are caused by oral biofilms; dental caries, gingivitis, periodontitis, peri-implantitis, and pulpitis are biofilm-related diseases. In health, the tooth pulp has a sterile interior [11]; being enclosed in mineralized tissue representing an extraordinarily isolated and well-protected environment in the human body [12]. The bacterially-derived infection of the dental pulp, namely pulpitis, occurs through the contact between pulp tissue and oral bacteria as a result of deep caries, dental trauma, periodontal pockets or iatrogenically-induced microleakage through insufficient restorations [11,13]. In consequence, the pulp loses its vitality because of the penetration of microorganisms and diverse microbial irritants through the dentin tubules. To prevent the spread of the infection to the adjacent tissues (possibly resulting in osteonecrosis, osteomyelitis, or disseminating endodontic infections) a local periapical lesion develops [14]. Endodontic infections aetiologically correlate with endodontic biofilms existing as intracanal-, external root-, and periapical biofilms [15]. Earliest reports showed that the intracanal flora consisted of cocci, rods, filamentous organisms, and spirochetes, forming clusters on dentinal walls of the root canal with a visible palisade structure [16]. Recent authors reported that the intraradicular biofilms in apical parodontitis could be found in the apex of the root canals, ramifications and isthmuses, on dentin walls and the outer root surface [17,18]. Microorganisms can penetrate the dentin tubules, and because of the presence of unmineralized collagen on the surface of the tubule itself, the initial colonizers can adhere to these, build the biofilm and persist even in treated root canals [13].

The pulpitis-related endodontic biofilm is mainly composed of caries-related bacteria, whereas the invasion of microorganisms through the dentinal tubules results in alterations in the biofilm composition, which is characterized by the dominance of Gram-negative anaerobic rods and proteolytic bacteria [11]. The primary intraradicular infections are caused by Gram-negative anaerobic rods of *Prevotella* sp. and *Porphyromonas* sp., the periodontal pathogen *Tannerella forsythia*, asaccharolytic obligate Gram-negative *Dialister* sp., *Fusobacterium* sp. and Gram-negative bacteria with periplasmic flagella such as *Treponema* sp. [12]. Besides this, primary infections also comprise Gram-positive anaerobic rods, Gram-positive cocci that can persist endodontic treatment (*Parvimonas micra*, *Streptococcus* sp., *Enterococcus faecalis*), Gram-negative microaerophilic rods (*Campylobacter* sp.) and several uncultivated phylotypes as well as fungi, archaea, and viruses [16]. Secondary infections can be caused by reinfection, remnant infection or recurrent infection in previously root canal treated teeth. Secondary infections are characterized by the persistence of enterococci, streptococci, lactobacilli, actinomyces, fungi and *E. faecalis* [19]. *Bacteroides-*like species and *Dialister* sp. were detected in asymptomatic endodontic infections associated with chronic periradicular lesions [20], whereas obligate anaerobes *Fusobacterium nucleatum, P. intermedia, Dialister pneumosintes* were found in symptomatic infections clinically diagnosed as acute abscesses [20]. Cultivation of isolates from root canals with necrotic pulps and failed endodontic treatment showed anaerobic or microaerophilic bacteria in 70% of cases, mainly containing *P. micra* (formerly *Peptostreptococcus micros)*, *Fusobacterium necrophorum*, *F. nucleatum*, *Prevotella intermedia/nigrescens*, *Porphyromonas gingivalis,* and *Porphyromonas endodontalis* [21].

Due to the importance of endodontic biofilms for oral health, there is a need of a multispecies biofilm model that mimics the in situ endodontic biofilms and enables a realistic and efficient investigation of new endodontic irrigants and antimicrobial therapies. For these purposes, we modified the ten-species subgingival Zurich biofilm model [22,23,24] and added a total of six bacterial species frequently found in endodontic infection (primary, secondary). We applied the batch culture approach, which was first described in 2001 [25], and is based on the biofilm model of supragingival plaque [24,26,27]. As about 50% of bacteria in the oral cavity are uncultivable and culture method only provides information about living cells [28,29,30], in the current study, a PCR-based 16S rRNA gene assay [31] was used for detection and quantification of bacterial species within the endodontic-like biofilms. Additionally, the endodontic-like biofilms were visualized using fluorescence in situ hybridization (FISH) and confocal laser scanning microscopy (CLSM). To the best of our knowledge, endodontic-like multispecies biofilms using hydroxyapatite as well as dentin as substrata were formed in vitro for the first time in this study. Based on this study, future investigations on endodontic infections can rely on this newly established endodontic-like multispecies biofilm model.

## 2. Methods

### 2.1. Multispecies Endodontic-Like Biofilm Formation

**Figure 1:**
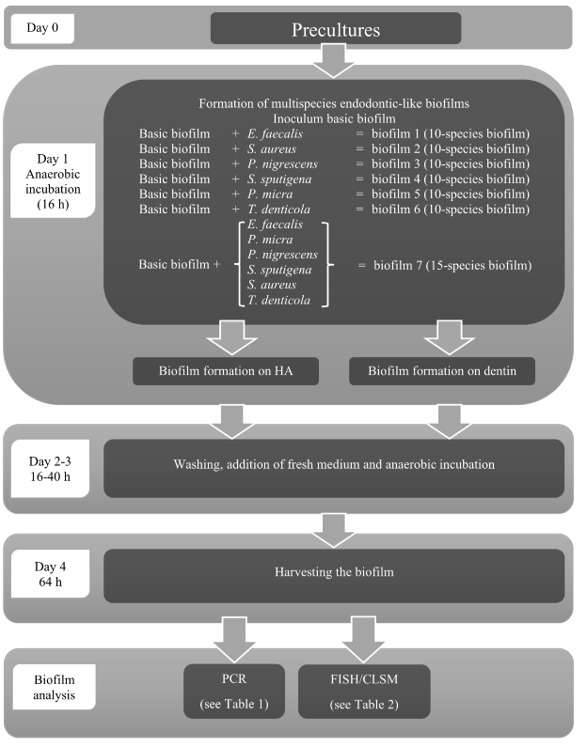
Endodontic-like biofilm formation and analysis using PCR and FISH/CLSM.

The nine-species basic biofilm used in this study (subsequently called “basic biofilm”) was produced according to the protocol of the standard ten-species subgingival in vitro biofilm [24] using a modified procedure described elsewhere [32]. In brief, this “basic biofilm” contained the following species: *Actinomyces oris* (OMZ 745), *Veillonella dispar* ATCC 17748^T^ (OMZ 493), *Fusobacterium nucleatum* (OMZ 598), *Streptococcus anginosus* ATCC 9895 (OMZ 871), *Streptococcus oralis* SK248 (OMZ 607), *Prevotella intermedia* ATCC 25611^T^ (OMZ 278), *Campylobacter rectus* (OMZ 388), *Porphyromonas gingivalis* ATCC 33277 (OMZ 925), and *Tannerella forsythia* ATCC 43037 (OMZ 1132). In order to establish an endodontic-like in vitro biofilm, the following bacterial strains were added to this “basic biofilm” (Figure 1): *Enterococcus faecalis* ATCC 29212 (OMZ 422) (biofilm 1), *Staphylococcus aureus* ATCC 2783 (OMZ 143) (biofilm 2), *Prevotella nigrescens* ATCC 33563 (OMZ 313) (biofilm 3), *Selenomonas sputigena* ATCC 35185 (OMZ 527) (biofilm 4), *Parvimonas micra* ATCC 33270^T^ (OMZ 518) (biofilm 5), or *Treponema denticola* ATCC 35,405 (OMZ 661) (biofilm 6). Finally, a 15-species endodontic-like biofilm was established containing the “basic biofilm” and the six aforementioned additional species (biofilm 7). The “basic biofilm” and biofilms 1–7 were numbered according to their composition (Figure 1) and were grown both on hydroxyapatite (HA) and dentin discs to simulate multispecies biofilm growth and spatial distribution on enamel and dentin.

All strains, except for *T. forsythia* and *T. denticola*, were maintained on Columbia blood agar. *Tannerella forsythia* and *T. denticola* were maintained in *T. forsythia* medium, containing per liter solution: 37 g brain-heart-infusion, 10 g yeast extract, 1 g cysteine, 5 µL/mL hemin, 20 µL/mL N-acetylmuramic acid, 2 µL/mL menadione and 5% horse serum. Prior to the onset of biofilm experiments, all strains were transferred into adequate liquid media (mFUM [33], BHI and *T. forsythia* medium) and incubated anaerobically at 37 °C for two cycles of precultures (16 h and 8 h, respectively). Prior to biofilm inoculation, all strains were adjusted to a defined optical density (OD_550_ = 1.0) and mixed in equal volumes. Biofilms were cultivated in 24-well polystyrene cell culture plates on sintered hydroxyapatite (HA; Ø 9 mm, Clarkson Chromatography Products, Inc., South Williams-port, PA 17702, USA) and dentin discs (Ø 7 mm, bovine teeth) that had been preconditioned (pellicle coated) for 4 h, with shaking (95 rpm) in 0.8 mL saliva (whole unstimulated saliva, pooled from individual donors [32], 1:2 diluted with sterile 0.25% NaCl solution; for the preparation of batches of pooled, processed, and pasteurized saliva, see Guggenheim et al. [25]. The pellicle-coated discs were equilibrated for 45 min at 37 °C in the anaerobic chamber in 1.6 mL growth medium (containing 960 μL undiluted saliva, 160 μL fetal bovine serum (FBS), and 480 μL mFUM + 0.3% glucose. Finally, the 200 μL bacterial suspension, consisting of equal volume and density (adjusted OD550 = 1.0) of each strain was given to each well, and the biofilm was incubated for 64 h under anaerobic conditions. At 16 and 40 h, the discs were washed 3 times with 2 mL of 0.9% NaCl (two dippings each) and transferred to fresh media in the 24-well plate. After 64 h the discs were washed again as previously and either proceeded to staining and confocal laser scanning microscopy (CLSM) or transferred into a 50 mL Falcon tubes with 1 mL of physiological NaCl and vortexed for 3 min in order to remove the biofilm from the discs, prior to the transfer to 5 mL Falcon tube and sonication at 30 W for 5 s (Sonifier B-12, Branson Ultrasonic, Urdorf, Switzerland). Then, the harvested biofilm suspension was prepared for quantification by real-time quantitative PCR (qPCR). For fluorescence in situ hybridization (FISH) and CLSM analyses biofilms were fixed in 1 mL of 4% paraformaldehyde + RNase inhibitor (RNAi) for two hours at 4–8 °C (Figure 1).

### 2.2. Biofilm Quantification Using Quantitative Real-Time PCR (qPCR)

The DNA was isolated from harvested biofilm samples and individual strains for standard curves using the GenEluate bacterial genomic DNA kit (Sigma-Aldrich, Saint Louis, Missouri, USA) according to the manufacturer’s recommendations including the pretreatment steps for Gram-positive bacteria with slight modifications. The pretreatment lysis step was expanded from 30 min to 1 h (lysozyme, mutanolysin, and lysostaphin) and the lysis step with proteinase K from 10 min to 20 min. The extracted DNA was eluted twice in 60 μL preheated nuclease-free water. The amount of the isolated DNA was determined using a spectrophotometer NanoDrop ND-1000 (Thermo Fisher Scientific, Waltham, Massachusetts, USA). The quantification of the individual bacteria templates in biofilm samples was generated by using external standard curves. The standard curves were created by defined concentrations from 10 ng to 0.00001 by 10-fold serial dilutions. The logarithm of the corresponding quantification cycle values was used to obtain a linear regression. The theoretical cell numbers of each organism in the samples were converted from the obtained Cq values using theoretical genome weight.

The SYBR Green-based detection was conducted to quantify bacteria in biofilm samples with the primers listed in Table 1. The quantitative PCR was carried out using the 2xSYBR^®^ Green PCR Master Mix (Thermo Fisher Scientific, Waltham, Massachusetts, USA) with a final reaction volume of 15 μL, containing 7.5 μL of SYBR^®^ Green PCR Master Mix, 6 μL sample DNA (undiluted, 1:10 and 1:100 diluted, respectively) and 1.5 μL of primer mix (final concentration 0.5 μM each). The qPCR assays were performed on a One Step Plus Real-Time PCR System (Applied Biosystems, Foster City, California, USA); samples were incubated initially 10 min at 95 °C, then 40 cycles of 15 s at 95 °C and 1 min at 60 °C.

For the quantification of *S. sputigena*, *P. micra*, *S. aureus* and *P. nigrescens* the microbial DNA qPCR assays were used and conducted according to the manufacturer’s protocol (Qiagen Instruments, Hombrechtikon, Switzerland; Cat. no. BPID00305AR, BPID00260AR, BPID00314A, and BPID00280AR, respectively).

### 2.3. Fluorescence in Situ Hybridization (FISH)

After fixation, discs were washed in 500 μL 0.9% NaCl + RNase Inhibitor and dabbed off on a paper towel. Pre-treatment of Gram-positive bacteria occurred as described before [23] within 1 mg/mL lysozyme solution in 0.1 M Tris-HCl, pH 7.5, 5 mM EDTA for 8 min at room temperature (RT). To permeabilize the cell walls accordingly *Staphylococcus aureus* cells needed a longer and stronger pre-treatment with 10 mg/mL lysozyme for 50 min at 37 °C and additionally with 20 µg/mL lysostaphin for 5 min at RT both in the same buffer as described previously. Pre-hybridization in 500 μL of proportionate hybridization buffer (Table 2) for 15 min at 46 °C. Immediately thereafter, the discs were transferred in extra wells with 370 μL preheated appropriate probes in the corresponding hybridization buffer (Table 2). The discs were hybridized for 4 h at 46 °C, then immersed in 2 mL preheated washing buffer and incubated for 45 min at 48 °C. Total DNA was stained with 15 µM Syto 59 (Thermo Fisher Scientific, Waltham, Massachusetts, USA) in nanopure water for 30 min or with 0.5 µg/mL DAPI (SERVA Electrophoresis GmbH, Heidelberg, Germany) in nanopure water for 5 min at room temperature. All incubations with fluorescent dyes were performed in the dark. Discs were embedded upside down on chamber slides in a matching drop of Mowiol and stored for at least 24 h before microscopic examination.

### 2.4. Confocal Laser Scanning Microscopy (CLSM)

CLSM was conducted using a Leica TCS SP5 microscope (Leica Microsystems, Wetzlar, Germany) provided by the Centre for Microscopy and Image Analysis of the University of Zurich. For the imaging of the biofilms on hydroxyapatite and dentin discs, the slightly modified procedure, as described before [38], was performed. Briefly, the used lasers were a UV laser at 405 nm excitation, an Argon laser at 488 nm excitation, a DPSS diode laser at 561 nm, and a Helium-Neon laser at 594 nm and 633 nm excitation. Furthermore, filters were adjusted at 430–470 nm to detect DAPI, at 500–540 nm for FITC, at 570–600 nm for Cy3, at 610–640 nm for ROX, and at 660–710 nm for Cy5 and Syto 59. Biofilms were scanned sequentially in steps of 1 μm thickness. Finally, the images were processed using Imaris 8.3 (Bitplane, Zurich, Switzerland).

### 2.5. Statistical Analysis

Within the three independent experiments with basic biofilm and additions of endodontic species, every group was represented in triplicate biofilm cultures. As a result, statistical analysis was performed on nine individual data points, coming from the nine individual biofilm cultures per experimental group. Two-way analysis of variance (ANOVA) was used to analyze the difference in bacterial cells per biofilm between the control group (standard nine-species biofilm) and the six additions of endodontic strains. Tukey’s multiple comparisons test was used for correction. Furthermore, the statistical comparison was performed between the number of cells per biofilm on hydroxyapatite discs and dentin discs, respectively. Missing values were ascribed the lowest detection limit value of the assay to allow for logarithmic transformation. Statistics have been implemented using GraphPad Prism (version 7) with the intent of comparing the species’ total cell counts within the different biofilm formations (significance level *p* < 0.05).

## 3. Results

### 3.1. The Addition of Endodontic Pathogens Induced Significant Changes in Cell Counts within Endodontic-Like Biofilms on HA

For this study a slightly modified in vitro subgingival biofilm described by Guggenheim et al. [24] was used and in the following is referred to “basic” nine species subgingival biofilm. This “basic” subgingival biofilm consisted of *Actinomyces oris*, *Veillonella dispar*, *Fusobacterium nucleatum*, *Streptococcus anginosus*, *Streptococcus oralis*, *Prevotella intermedia*, *Campylobacter rectus*, *Porphyromonas gingivalis*, and *Tannerella forsythia*. In order to guarantee reproducibility of the new established biofilms, all assays were conducted three times in triplicates.

**Figure 2:**
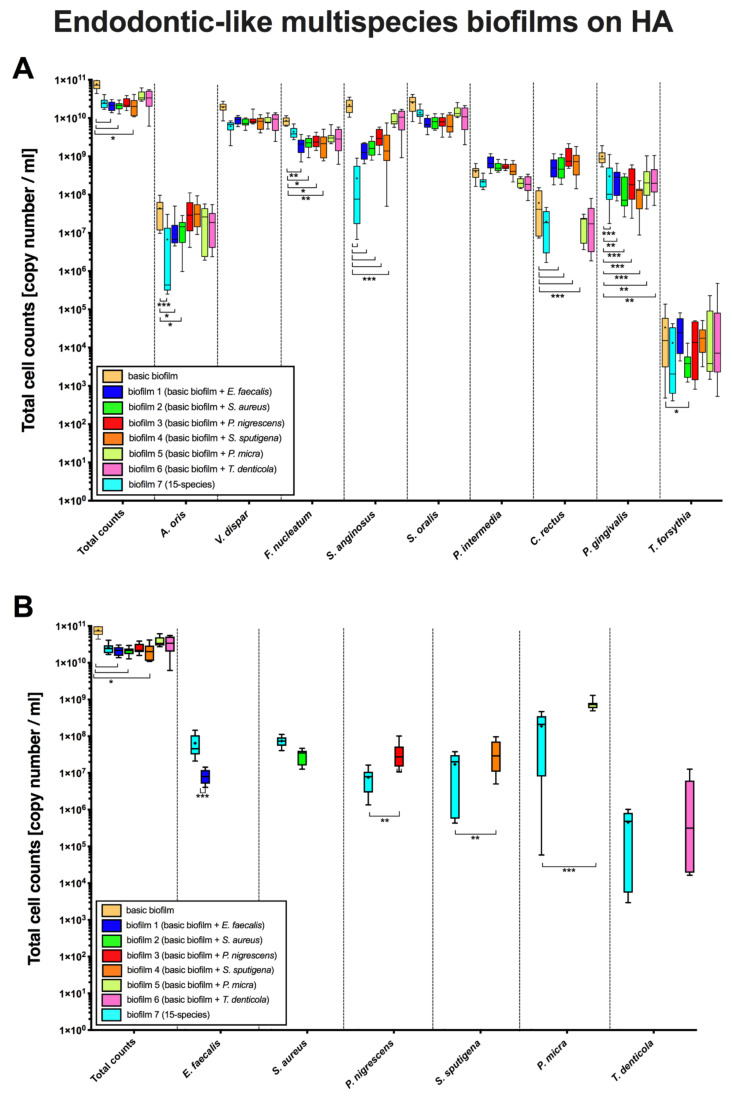
Boxplots demonstrating cell counts per endodontic-like biofilm on pellicle-coated hydroxyapatite discs after analysis by qPCR. To form endodontic-like multispecies biofilms, a total of six bacterial species were added separately to a “basic” nine-species subgingival biofilm. *x*-axis of panal (**A**) shows the strains of the “basic biofilm” (in the first column total counts (beige) as a control group are shown), while *x*-axis of panal (**B**) shows in the first column total counts (beige) again, as well as the strains of the endodontic species (*E. faecalis* (blue), *S. aureus* (dark green), *P. nigrescens* (red), *S. sputigena* (orange), *P. micra* (light green) and *T. denticola* (pink)). Statistically significant differences between the biofilm with additional strains and the control group (“basic biofilm” or endodontic-like biofilm) is marked with 1–4 asterisks (* *p* < 0.05; ** *p* < 0.01; *** *p* < 0.001). The internal line represents the median; the whiskers indicate minimum and maximum. The *p* values (*p* ≤ 0.05) of the significantly different data are provided. Data derive from three independent experiments, each represented in triplicate biofilm cultures (*n* = 9).

Box plots in Figure 2 demonstrate cell counts per endodontic-like biofilm on pellicle-coated HA discs after analysis by qPCR. To form endodontic-like multispecies biofilms, a total of six endodontic pathogens were separately added to a “basic” nine-species subgingival biofilm (see Methods, Figure 1).

The total cell counts within the ”basic biofilm” were significantly lower compared to the number of cells per biofilm (Figure 2A, 1st column) when the biofilms contained *E. faecalis* (biofilm 1, *p* = 0.032)*, S. aureus* (biofilm 2, *p* = 0.031) or *S. sputigena* (biofilm 4, *p* = 0.020), respectively. These findings indicate that the growth of “basic species” was affected by the addition of other endodontically-relevant species. On this basis, a significant reduction of *A. oris* (*p* < 0.0001), *S. anginosus* (*p* < 0.0001) and *P. gingivalis* (*p* = 0.0002) counts was observed in endodontic-like biofilms (containing all 15 species; biofilm 7) compared to the “basic biofilm”. The *A. oris* counts were significantly lower in biofilms containing *E. faecalis* (biofilm 1; *p* = 0.037) and *S. aureus* (biofilm 2; *p* = 0.031) than in “basic biofilm”. The *F. nucleatum* counts also significantly decreased in biofilms containing *E. faecalis* (biofilm 1; *p* < 0.005)*, S. sputigena* (biofilm 4; *p* < 0.0034), *S. aureus* (biofilm 2; *p* = 0.0154) and *P. nigrescens* (biofilm 3; *p* < 0.0496) compared to the “basic biofilm”. The *S. angingosus* counts decreased with the addition of *E. faecalis, S. aureus, P. nigrescens, and S. sputigena* compared to the “basic biofilm” (biofilms 1, 2–4; *p* < 0.0001) as well.

Contrarily to this, *C. rectus* counts substantially increased (*p* < 0.0001) in biofilms 1–4 when *E. faecalis, S. aureus*, *P. nigrescens*, and *S. sputigena* were added to the “basic biofilm”. On the other hand, composition of endodontic-like biofilms containing all 15 species (biofilm 7) caused a decrease in *P. gingivalis* counts compared to *P. gingivalis* counts in the nine-“basic biofilm”. Interestingly, the counts of *T. forsythia* decreased only when *S. aureus* was present (biofilm 2, *p* = 0.049). Regarding the species added to the “basic biofilm” a decrease of *E. faecalis* cells (biofilm 1, *p* < 0.0001) and an increase in *P. nigrescens* (biofilm 3, *p* < 0.001), *S. sputigena* (biofilm 4, *p* < 0.001) and *P. micra* (biofilm 5, *p* < 0.0001) counts was observed compared to the 15-species biofilm (biofilm 7).

### 3.2. The Bacterial Composition of Endodontic-Like Biofilms on Dentin Was Also Substantially Affected by the Presence of Endodontic Pathogens

**Figure 3:**
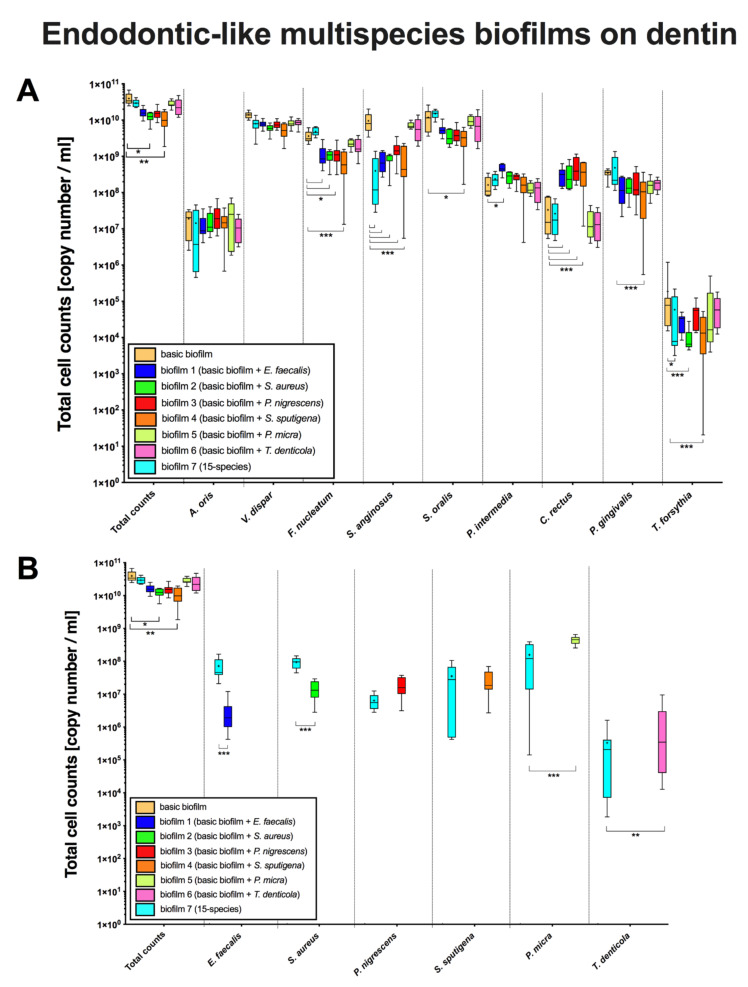
Boxplots demonstrating cell counts per endodontic-like biofilm on dentin discs after analysis by qPCR. To form endodontic-like multispecies biofilms, a total of six bacterial species were added separately to a “basic” nine-species subgingival biofilm. The *x*-axis of panal (**A**) shows the strains of the “basic biofilm” (in the first column total counts (beige) as a control group are shown), while the x-axis of panal (**B**) shows in the first column total counts (beige) again, as well as the strains of the endodontic species (*E. faecalis* (blue), *S. aureus* (dark green), *P. nigrescens* (red), *S. sputigena* (orange), *P. micra* (light green) and *T. denticola* (pink)). Statistically significant differences between the biofilm with additional strains and the control group (basic biofilm or all species biofilm) is marked with 1–4 asterisks (* *p* < 0.05; ** *p* < 0.01; *** *p* < 0.001). The internal line represents the median; whiskers indicate minimum and maximum. The *p* values (*p* ≤ 0.05) of the significantly different data are provided. The data were derived from three independent experiments, each represented in triplicate biofilm cultures (*n* = 9).

Box plots in Figure 3 demonstrate cell counts per endodontic-like biofilm on dentin discs after analysis by qPCR. To form endodontic-like multispecies biofilms, a total of six endodontic pathogens were separately added to a “basic” nine-species subgingival biofilm.

The total cell counts within the endodontic-like biofilm on dentin discs containing *S. sputigena* were significantly lower (biofilm 4, *p* = 0.011) compared to the number of cells per biofilm in the “basic biofilm” (Figure 3, 1st column). The total cell counts of *A. oris* and *V. dispar* in the “basic biofilm” did not differ from the total cell counts in the endodontic-like biofilms 1–6 (Figure 3A). However, *F. nucleatum* counts decreased for all strains (biofilms 1–3 *p* < 0.05; biofilm 4 *p* < 0.0001) except for *P. micra* and *T. denticola* (*p* = 0.950 and *p* = 0.746, respectively). Similar findings were obtained for cell counts of *S. anginosus* in biofilms 1–4 (*p* < 0.0001). Interestingly, the total cell counts of *F. nucleatum* and *S. anginosus* were precisely the same as on HA discs.

While the addition of *E. faecalis* on dentin discs (biofilm 1) did not affect the basic biofilm, a positive impact on the growth of *P. intermedia* on dentin discs could be observed (*p* < 0.05). As on HA discs, the addition of *E. faecalis* (biofilm 1), *S. aureus* (biofilm 2)*, P. nigrescens* (biofilm 3)*,* and *S. sputigena* (biofilm 4) affected the growth of *C. rectus* positively (*p* < 0.0001). The total cell counts of *P. gingivalis* was significantly lower (*p* < 0.0001) in biofilm 4 containing *S. sputigena* than in the “basic biofilm”. The addition of *S. aureus* (biofilm 2) and *S. sputigena* (biofilm 4) to the “basic biofilm” induced a substantial decrease (*p* < 0.0001) in *T. forsythia* counts. That is reflected by a significant decrease (*p* = 0.022) of *T. forsythia* counts in endodontic-like 15-species biofilm (biofilm 7) in comparison with the *T. forsythia* counts in the “basic biofilm”.

Regarding additional species there was a decrease in *E. faecalis* and *S. aureus* (*p* < 0.0001) counts and an increase in *P. micra* (*p* < 0.0001) and *T. denticola* (*p* < 0.0001) counts in biofilms 1, 2, 5, and 6, respectively, compared to counts of these species in the biofilm 7 on dentin discs.

### 3.3. Different Substrates Did Not Affect the Composition of the Endodontic-Like Multispecies Biofilms

**Figure 4:**
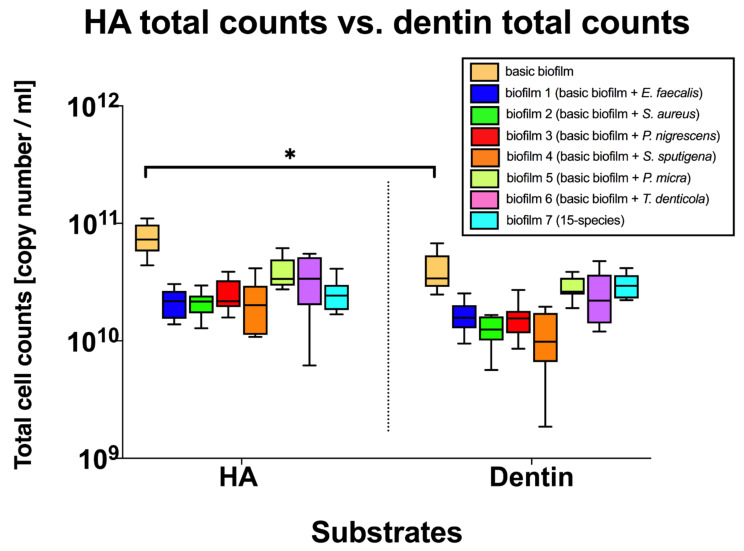
Boxplots demonstrating total cell counts per endodontic-like biofilm on HA and dentin discs after analysis by qPCR. To form endodontic-like multispecies biofilms, a total of six bacterial species were added separately to a “basic” nine-species subgingival biofilm. The *x*-axis shows endodontic-like biofilms on HA and dentin discs. Statistically significant differences between the total counts of the two substrates is marked with 1–4 asterisks (* *p* < 0.05). The internal line represents the median; whiskers indicate minimum and maximum. The *p* values (*p* ≤ 0.05) of the significantly different data are provided. The data were derived from three independent experiments, each represented in triplicate biofilm cultures (*n* = 9).

In the box plots in Figure 4 cell counts per endodontic-like biofilm on HA and dentin discs after analysis by qPCR are shown. Regarding total counts, only the “basic” subgingival biofilm showed a significant reduction of cell counts (*p* = 0.019) when grown on dentin, whereas total counts of the endodontic-like multispecies biofilms were not affected by the different substrates.

### 3.4. FISH/CLSM Reveals E. faecalis Aggregates and S. aureus Microcolonies within Endodontic-Like Biofilms

**Figure 5:**
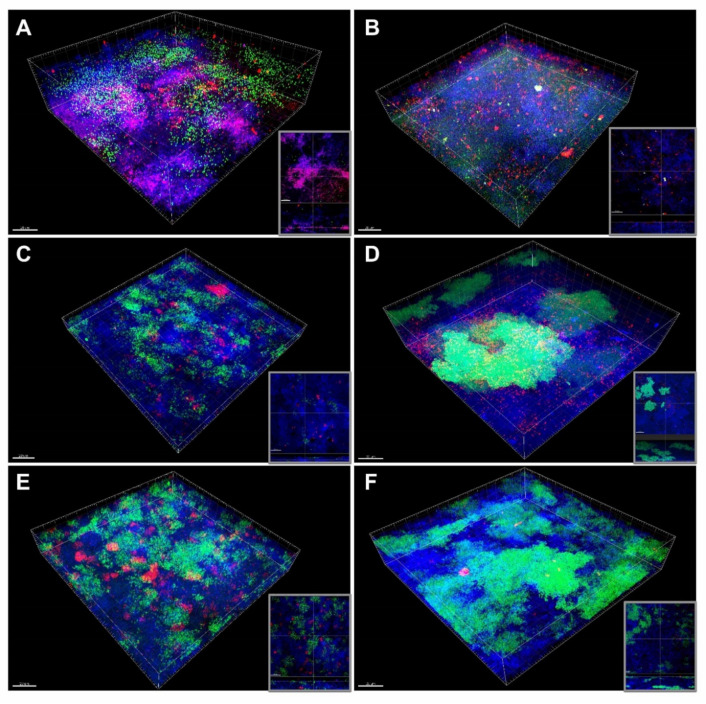
Confocal laser scanning microscopy (CLSM) 3D reconstructions and image stacks (insets) of endodontic-like biofilms grown on HA discs following fluorescence in situ hybridization (FISH) using FITC- and Cy3-labelled probes (see Table 2). To form endodontic-like multispecies biofilms, a total of six bacterial species were added separately to a “basic” nine-species subgingival biofilm. The resulting biofilms 1–6 contained additionally *E. faecalis* (**A**), or *S. aureus* (**B**), or *P. nigrescens* (**C**), or *P. micra* (**D**), or *S. sputigena* (**E**), or *T. denticola* (**F**). *Prevotella intermedia* appears green (FITC-labeled) and the newly added bacteria appear red (Cy3-labeled). Non-hybridized bacteria appear blue due to DNA staining (YoPro 59). Scale bar = 10 µm.

Figure 5 shows CLSM images of endodontic-like ten-species biofilms 1–6 grown on HA discs following FISH using FITC- and Cy3-labelled probes (see Table 2). *Enterococcus faecalis* (Figure 5A) cells seem build aggregates in the ten-species endodontic-like biofilm (biofilm 1). Figure 5B shows *S. aureus* situated on the bottom of the biofilm and forming microcolonies. *Prevotella nigrescens* (Figure 5C) and *P. micra* (Figure 5D) seem to be scattered throughout the biofilm. The same applies in respect of *S. sputigena* (Figure 5E) but forming larger aggregates more or less scattered throughout the biofilm. *Treponema denticola* (Figure 5F) seems to be spread in a low amount on the bottom of the biofilm 6.

**Figure 6:**
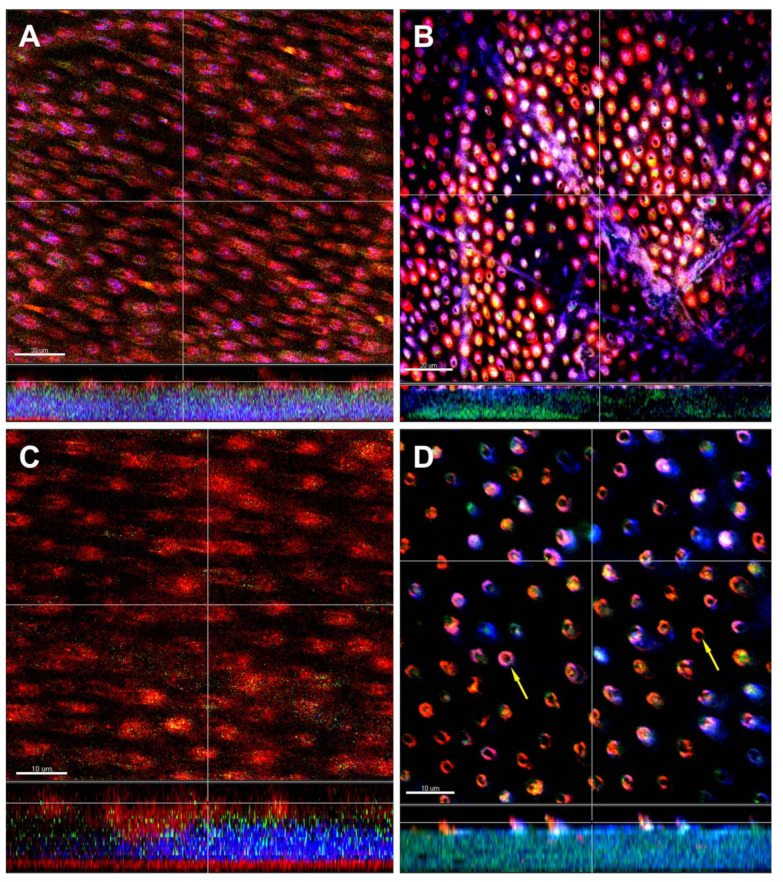
Confocal laser scanning microscopy (CLSM) image stacks of the basic nine species biofilm grown on dentin discs containing additionally *Enterococcus faecalis* (**A**), or *Staphylococcus aureus* (**B**), or *Selenomonas sputigena* (**C**), or *Prevotella nigrescens* (**D**). Due to FISH staining of biofilms using FITC- and Cy3-labelled probes (see Table 1), *Prevotella intermedia* appears green (FITC-labeled) and the newly added bacteria appear red (Cy3-labeled). Non-hybridized bacteria appear blue due to DNA staining (YoPro 59). Images were taken at the biofilm base showing dentinal tubules. The arrows indicate bacteria adhered in tubules. Scales = 20 µm (**A**,**B**) and 10 µm (**C**,**D**).

Figure 6 shows CLSM images of endodontic-like ten-species biofilms 1–6 grown on dentin discs following FISH using FITC- and Cy3-labelled probes (see Table 2) and highlights the fact that dentin tubules are colonized by bacteria. Figure 6A,C show dentin tubules filled with cells of *E. faecalis* and *S. sputigena*, respectively. *Prevotella intermedia* cells cannot be seen; it seems that they did not invade the dentin tubules. Figure 6B shows cells of *S. aureus* on the bottom of the biofilm at the interface with dentin tubules. Figure 6D clearly shows cells of *P. nigrescens* (arrows) within the dentin tubules.

**Figure 7:**
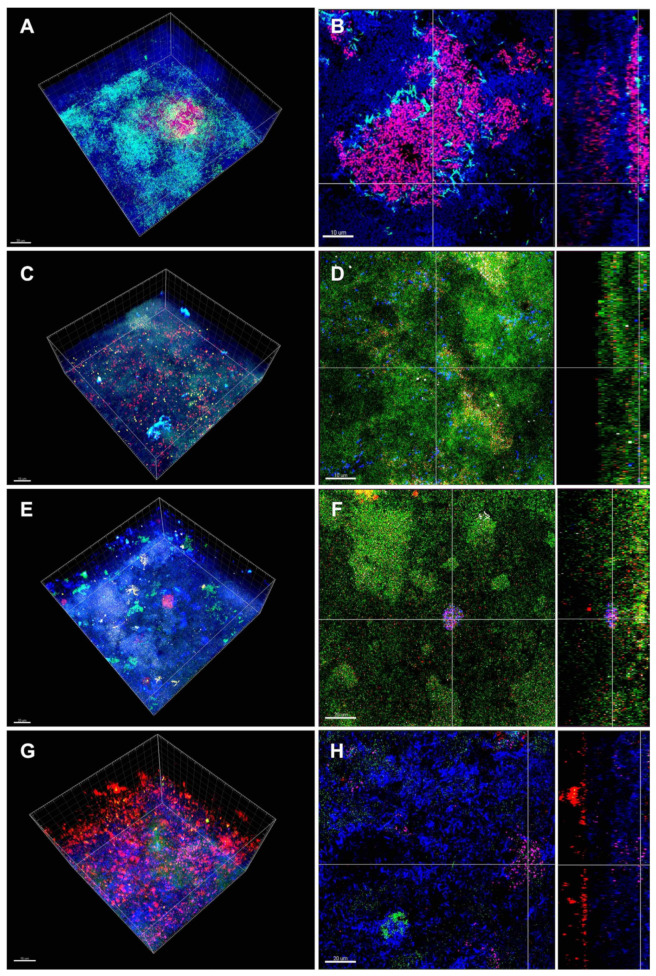
Confocal laser scanning microscopy (CLSM) 3D reconstructions of the endodontic-like 15-species biofilms grown on HA discs following fluorescence in situ hybridization (FISH). To form endodontic-like multispecies biofilms, a total of six bacterial species were added to a “basic” nine-species subgingival biofilm. The resulting endodontic-like 15-species biofilms contained *F. nucleatum* (light blue; FITC-labeled) and *P. intermedia* (red; Cy3-labeled) (**A**,**B**); *E. faecalis* (green; FITC-labeled), *P. micra* (red; Cy3-labeled), *S. aureus* (yellow; ROX-labeled) (**C**,**D**); *P. nigrescens* (green; FITC-labeled), *S. sputigena* (red; Cy3-labeled), *T. denticola* (white; ROX-labeled) (**E**,**F**); *P. gingivalis* (green; FITC-labeled), *T. forsythia* (red; Cy3-labeled), *P. intermedia* (purple; ROX-labeled), *F. nucleatum* and *C. rectus* (blue; Cy5-labeled) (**G**,**H**). In images (**A**–**F**), non-hybridized bacteria appear blue due to DNA staining (YoPro 59). Scale bar = 10 µm (**A**–**E**,**G**) or 20 µm (**F**,**H**).

Figure 7 shows CLSM images of endodontic-like 15-species biofilms (biofilm 7) grown on HA discs following FISH. Figure 7A,B show *P. intermedia* bacteria forming aggregates in the middle of the biofilm surrounded by *F. nucleatum*. *Enterococcus faecalis*, *P. micra* and *S. aureus* grow homogenously scattered throughout the biofilm (Figure 7C,D). Figure 7E,F show *P. nigrescens, S. sputigena, T. denticola* forming larger aggregates. Interestingly, aggregates of *P. nigrescens* and *T. denticola* could be observed in immediate vicinity to each other. Finally, many FISH-labeled bacteria, namely *P. gingivalis, T. forsythia, P intermedia, F. nucleatum,* and *C. rectus,* were visualized in the biofilm 7 (Figure 7G,H). It seems that *P. gingivalis* was located at the top of the biofilm, while *T. forsythia* was situated on the bottom of the biofilm. *Prevotella intermedia*, could be visualized in the intermediate layer of the biofilm, together with *F. nucleatum* and *C. rectus* (Figure 7H).

## 4. Discussion

In this study, new endodontic-like multispecies biofilm models (ten-species biofilms 1–6, 15-species biofilm 7) were formed for the first time and the role of different substrates on biofilm formation was investigated. Mixed-species biofilms are the dominant form in nature and are also prominent in the oral cavity as more than 700 microbial species inhabit this environment [43]. These biofilms resemble multi-cellular organisms and are characterized by their overall metabolic activity upon multiple cellular interactions. The development of a mixed-species biofilm is influenced by its species and by interactions between these microorganisms. Cell–cell communication or quorum sensing mediated by signal molecules can affect such interactions within mixed-species biofilms e.g., by altering gene expression that can result in synergistic or antagonistic interbacterial interactions [44,45,46]. For instance, two bacterial species that are involved in periodontits and endodontitis, *Treponema denticola* and *Porphyromonas gingivalis*, displayed synergistic effects in in vitro biofilm formation [47]. Competition among species in a mixed biofilm can be influenced by environmental conditions known e.g., by production of antistreptococcal bacteriocins [48]. The necessity of endodontic multispecies biofilm models to study the complex interspecies interactions in endodontic diseases has been already underlined in the literature so far [19]. Supragingival, subgingival, and endodontic biofilms constitute a very complex, organized entity and it is difficult, if not impossible to duplicate their characteristics in in vitro experiments. The complexity is not only related to the nature of the biofilm, but also to the complex anatomy, which houses tissue along with biofilms [19]. Biofilm models developed in Zurich are standing out due to their exceptional reproducibility for applications with direct or indirect impact on prophylactic dentistry such as spatial arrangement and associative behavior of various species in biofilms [24,25,26,32,49,50,51,52,53,54]. The overall physiological parameters of multispecies biofilms can be measured quite accurately, but it is still impossible to assess the multitude of interactions taking place in such complex systems [50]. In this study, an endodontic-like multispecies biofilm was used containing representative organisms found in supragingival, subgingival and endodontic-like biofilms to enable camparison of endodontic-like biofilm formation between enamel and dentin surfaces. To form endodontic-like multispecies biofilms, a total of six endodontic pathogens were separately added to a “basic” nine-species subgingival biofilm. The multispecies biofilm formation on pellicle-coated HA and dentin discs was compared showing strong similarities in regard with the cell counts per biofilm. Regarding total counts neither of the endodontic-like biofilms 1–7 showed a significant difference on the two substrates, only the “basic” nine species subgingival biofilm showed reduced total counts on dentin. A study by Jung et al. [55] showed that bacterial colonization was higher on dentin than on enamel, however, this was an in situ study and investigated initial colonization.

By adding six different strains of bacteria one by one, we observed different effects of the added strains on the “basic biofilm”. For example, the addition of *E. feacalis* affected negatively the growth of *A. oris, F. nucleatum, S. anginosus* and *P. gingivalis* in biofilm 1 on HA-discs. However, *E. faecalis* affected negatively the growth of *F. nucleatum* and *S. anginosus* on dentin discs. Previous clinical studies showed a significant relation between the presence of *E. faecalis* in asymptomatic primary endodontic infections [56], although *E. faecalis* is known for persisting in endodontic infections associated with root-filled teeth [56,57]. This microorganism was also found as a monospecies infection even after intracanal medication. The high persistence of *E. faecalis* can be attributed to its natural adaption to adverse ecological conditions in the root canals [58,59] or to the formation of biofilms [60,61]. A previous report by Chávez de Paz et al. [62] showed that different *E. faecalis* strains differ in their capacity to produce different proteases depending on their origin and to suppress the growth of other species in multispecies biofilms [59]. In the present study, we used the vancomycin-sensitive strain *E. faecalis* ATCC 29212, which serves as a representative control strain in many in vitro trials, because of its availability in our lab. Regarding the *E. faecalis*-associated suppression of growth of other species within a biofilm, our findings are in line with the results of a previous research using the oral strain OGRF1 [59]. In our study, *E. faecalis* ATCC29212 seems to have suppressed the growth of other oral species within the basic biofilm on HA-discs; the total cell counts within the biofilm 1 containing *E. faecalis* were significantly lower compared to the cell counts in the ”basic biofilm” without *E. faecalis*.

Furthermore, the addition of *E. faecalis* affected negatively the growth of *A. oris, F. nucleatum, S. anginosus* and *P. gingivalis* in biofilm 1 on HA-discs. A similar finding regarding *A. oris* was highlighted by Thurnheer and Belibasakis [40] after studying the incorporation of *E. faecalis* into supragingival biofilms on HA-discs. In a previous study by Ran et al. [63], it was observed that *E. faecalis* cells were able to form biofilms despite the nutrient reduction in the local microenvironment. Moreover, the hydrophobicity of *E. faecalis* cells increased under starvation conditions, and the biofilm-related gene transcription was triggered by oxygen/nutrient deprivation. Our findings seem to be in line with this study concerning nutrient supply. In specific, *E. faecalis* cell counts showed an increase in the 15-species biofilm (biofilm 7) compared to the 10-species biofilm (biofilm 1) both on HA discs and dentin discs. This finding is illustrated in Figure 7C,D, which show a high number of *E. faecalis* cells within the biofilm 7.

Likewise, the addition of *S. aureus* to the “basic biofilm” affected negatively the growth of *A. oris, F. nucleatum, S. anginosus*, *P. gingivalis* and *T. forsythia* on HA-discs. Interestingly, *S. aureus* yielded similar effects on *F. nucleatum, S. anginosus* and *T. forsythia* when grown on dentin discs. In previous studies *S. aureus* was identified in samples from infected teeth root canals associated with endodontic abscesses [64], as well as in samples from healthy periodontal tissues representing a source for systemic infections [65]. Previous research by Thurnheer and Belibasakis [32] on the growth of *S. epidermidis* on HA and titanium in a biofilm model for peri-implantitis showed that *S. aureus* possessed the trait to outcompete other oral bacterial species. To confirm this finding, Makovcova et al. [66] also noticed that there was a general competition between *S. aureus* and Gram-negative bacteria *in vitro*. The group stated that *S. aureus* grew in smaller clusters in mixed-species biofilms than in *S. aureus* monospecies biofilms. However, we showed the opposite effect of a large bacterial consortium (15-species versus 10-species biofilms) on *S. aureus* on dentin discs (Figure 3). Particularly, *S. aureus* showed higher growth in 15-species-biofilms than in 10-species-biofilms. This outcome confirms the synergistic interactions between *S. aureus* and other species in polymicrobial biofilms as already described by Giaouris et al. [67].

In contrast to *S. aureus, Prevotella nigrescens* counts decreased in 15-species-biofilm compared to 10-species biofilm (biofilm 3), both on HA discs and dentin discs. *Prevotella nigrescens* is a black-pigmented bacterium often detected in endodontic infections. To discriminate it from *P.* intermedia SDS-PAGE was used [68,69]. Previously, *P. intermedia* was for decades supposed to be the most frequently detected species associated with endodontic infections [68,69]. *Prevotella nigrescens* belongs to Gram-negative anaerobic bacteria and together with *P. intermedia* and *P. gingivalis* is associated with necrotic pulp tissue [70]. *Prevotella nigrescens* subsists on glucose [71] and its decrease may be related to the relatively higher supply of glucose in biofilm 3 compared to biofilm 7. The glucose catabolism of *P. nigrescens* may induce a decrease in the pH of the biofilm [71]. Opposed to *P. nigresens, P. gingivalis* is an asaccharolytic bacterium whose growth does not depend on fluctuations of glucose but on the supply of amino acids and haemin [72]. In the absence of glucose both *P. intermedia* and *F. nucleatum* produced acid-neutralizing metabolites leading to increased pH, as shown earlier in an in vitro study by Takahashi et al. [73]. It was observed that a basic pH, as can be found in in vivo subgingival biofilms, supported the growth of *P. gingivalis* [73]. Though, in the current study, in a glucose-containing medium, the addition of *P. nigrescens* to the “basic biofilm” affected negatively the growth of *P. gingivalis* on HA-discs. Thus, the low growth of *P. gingivalis* might have been negatively influenced by the pH decrease reinforced by the addition of *P. nigrescens.* Moreover, there was an overall decrease of the *P. gingivalis* counts in biofilms 1–6 compared to the *P. gingivalis* counts in “basic biofilm” on HA discs. In the same way, there was a decrease of *P. gingivalis* counts in biofilm 7 (all species) compared to “basic biofilm”. These findings may be related to the pH decrease because of the presence of glucose. Contrarily to glucose, haemin [72] and amino acids are the nutrients for *P. gingivalis* and these are running out faster in a bigger consortium of species. Therefore, these results may rather be based on the depletion of resources in biofilms 1–7 compared to “basic biofilm”.

In a similar manner as *P. nigrescens, Selenomonas sputigena* negatively affected the growth of *F. nucleatum, S. anginosus* and *P. gingivalis* on HA-discs. On dentin discs, the same effects were observed for the same species, including *S. oralis* and *T. forsythia*. Furthermore, the addition of *S. sputigena* negatively affected the total bacterial cell counts in the endodontic-like biofilm on dentin discs compared to the counts in biofilm 4. These results might correspond to the findings by Rocas et al. [74] who detected *S. sputigena* in symptomatic cases of endodontic infections associated with sinus tract. After all, *Selenomonas sputigena* was identified in a different community than e.g., *Streptococcus* spp., *F. nucleatum*, *P. gingivalis*, and *T. forsythia* [74].

*Tannerella forsythia* had the greatest difficulty to establish in the “basic biofilm” as well as in biofilms 1–6 on HA and on dentin discs. These results correlate with the findings made by Guggenheim et al. [24] using a subgingival in vitro biofilm model. Furthermore, Zhu et al. [75] pointed out similar observations regarding the counts of *T. forsythia* grown in a flow cell system together with *T. denticola* and *P. gingivalis*. Given the fact that *T. forsythia* grew well as a single-species in a planktonic state, our findings might be confirming *T. forsythia*’s general difficulties to establish in polymicrobial in vitro biofilm models.

Together with *P. gingivalis* and *T. forsythia, Treponema denticola* constitutes the red-complex bacteria and it has been detected in necrotic pulps associated with swelling caused by primary endodontic infections [76]. Previous research suggested that the strong synergistic association between *T. denticola* and *P. gingivalis* based on the motility of *T. denticola* [75]. In fact, it was shown that fibrilin binds to dentilisin of *T. denticola* enabling the coaggregation between these two species inside of periodontal pockets resulting in an up-regulation of the fibrilin gene [77]. CLSM images of a 10-species subgingival biofilm model by Ammann et al. [23], showed *T. denticola* growing loosely in the top layer along with *P. gingivalis*. However, our images showed *T. denticola* situated in the intermediate layer and on the bottom of the biofilm (Figure 5F), building star-shaped clusters in proximity to *P. nigrescens* (Figure 7C) (*P. gingivalis* cannot be distinguished from the other bacteria in this Figure). Surprisingly, the addition of *T. denticola* to the “basic biofilm” negatively affected only the growth of *P. gingivalis* on HA-discs (biofilm 6). A previous inquiry on metatranscriptome demonstrated that the gene expression of *T. denticola* differed dramatically in vitro from in vivo conditions [77]. This finding may explain the absence of in vitro synergetic interactions between these two species. However, as mentioned before, the addition of the endodontic strains to the “basic biofilm” on HA discs (biofilms 1–5) had a similar effect on *P. gingivalis*. Previous research by Neilands et al. [78] showed that *P. micra* enhanced the growth of *P. gingivalis* in 10% serum. This finding, however, could not be observed in the present study.

In addition to *P. gingivalis*, *Streptococcus anginosus* showed a similar behavior in biofilms 1–4 by addition of the endodontic species. Previous studies showed that *S. anginosus* depends on glucose and amino acids for its homeostasis and has a slow metabolism regarding recovery from nutrient deprivation [79]. Even if this is a survival strategy in the oral cavity where nutrients intake varies, this result might have been a disadvantage regarding the cell growth in this study. The growth of *S. anginosus* dropped significantly in the 15-species biofilm compared to the “basic biofilm” (both on HA- and dentin discs). Munson et al. [28] suggest that species with fast metabolism inhibit species with slow metabolism by the emission of metabolic products. This finding may explain the reduced cell counts of *S. anginosus* in our polymicrobial in vitro biofilm model containing 15 different species.

Only two of the “basic strains” were positively affected by addition of the new species, namely *C. rectus* both on HA and dentin discs and *P. intermedia* only on dentin discs. In previous studies, *C. rectus* was detected in primary endodontic infections associated with periradicular lesions [80]. Furthermore, *C. rectus* was positively associated with *P. endodontalis*, *P. micra*, *S. sputigena*, *F. nucleatum*, and *Actinomyces* sp. probably due to the production of growth factors, like formate [80]. This may explain the finding why the adddition of *E. faecalis*, *S. aureus*, *P. nigrescens* and *S. sputigena* enhanced the growth of *C. rectus* both on HA and on dentin discs.

In order to examine endodontic biofilm architecture, the biofilm of apical periodontitis of extracted teeth was analysed by Ricucci et al. [18]. The authors could not find a morphological pattern in this biofilm regarding the composition of bacteria (cocci, rods, filaments) and the amount of extracellular matrix and extent of the biofilm in the root canal. Examining the subgingival biofilm formation on natural teeth, Zijnge et al. [39] found 4 layers beginning with the early colonizers *Actionomyces* sp. in the basal layer. Periodontal Gram-negative pathogens like *P. gingivalis*, *P. intermedia*, *P. endodontalis, P.nigrescens* were found in the top layer and *Spirochetes* could be detected outside of the biofilm. Similary, our Figure 5D and Figure 7C show *P. intermedia,* together with the Gram-positive *P. micra,* established on the top of the biofilm (Figure 7G, 7H). *Fusobacterium nucleatum* that was detected in the intermediate layer previously [39], is a bridge-building microorganism [9] that facilitates the binding of initial colonizers with late colonizers such as *P. gingivalis*, *P. nigrescens* and *P. intermedia* [81,82]. Accordingly, in the present report, CLSM images show in the center of the biofilm aggregation of *P. intermedia* cells surrounded by cells of *F. nucleatum* (Figure 7A) and *C. rectus* (Figure 7H). Another CLSM image (Figure 6D) shows *P. nigrescens* at the bottom of the biofilm at the interface with dentin invading dentin tubules, while *P. intermedia* is homogeneously distributed throughout the biofilm (Figure 5, 6, 7), especially in the intermediate biofilm layer. Again, the cells of *T. forsythia* could be detected at the bottom of our multispecies biofilm model (Figure 7G, 7H). This was not in accordance with the spatiotemporal model of oral bacterial colonization and previous findings that pathogens like *T. forsythia* were mostly present as microcolonies in the top layer of biofilms [39].

In addition to *P. nigrescens*, other bacterial species such as *E. faecalis, S. aureus* and *S. sputigena,* were detected at the openings of dentin tubules (Figure 6). Jung et al. [55] used FISH/CLSM to visualize the colonization of dentin tubules by bacteria *in situ*. Previous research by Love [83] demonstrated in vitro the adhesion of *E. faecalis* on tooth roots in medium containing human serum. Interestingly, *E. faecalis* invaded dentin tubules by adhesion to exposed unmineralized collagen [83]. In another study, Sum et al. [84] showed that the adherence of *E. faecalis* to collagen varies and can be enhanced by chemical alteration of the dentin surface.

There are several limitations associated with the use of in vitro biofilms, such as the lack of a host defence system, necrotic tissue, innervation and living odontoblasts [85]. Using modern techniques, it is still not possible to say if the in vitro biofilms consist of the same extracellular matrix like the corresponding in situ biofilms [19]. There has been an attempt to mimic the environment within dental tubules and root canals using only human serum as a medium, but this condition decelerated the growth of *E. faecalis* [83]. Thus, there is a need for further studies on endodontic biofilms using diverse media in order to get even closer to in vivo conditions.

Even though CLSM is the method of choice for visualizing the biofilm matrix, it does not provide detailed information about the ultrastructure of biofilm because of the low magnification [86]. Thus, a combination of image data of different methods would provide a more accurate picture of a biofilm architecture than the FISH/CLSM alone [86,87]. A limitation of qPCR is that it amplifies all target DNAs, including that from non-viable cells. Amplification of DNA from dead cells could be inhibited by coupling qPCR with propidium monoazide [88,89]. Real-time qPCR has been described as the gold standard for RNA quantification, although the reproducibility and reliability of this method have been questioned so far [90,91]. The present study introduces a new endodontic-like multispecies biofilm. The findings of this study can be used in endodontic research for testing new antimicrobial agents and simulating endodontic flora for various endodontic applications. Furthermore, these findings enable researchers to test the effects of different antibiotics on endodontic biofilms under in vitro conditions. Further work is needed to depict the microscopic architecture of our endodontic-like multispecies biofilm model and to explore the complex interspecies interactions in endodontic disease. We also have to keep in mind that not only the species composition, but also the genetic expression can change in different biofilms. However, the study of the changes in the metatranscriptome of the biofilms was beyond the objectives of this work and is the focus of future studies.

In conclusion, the present study shows successful incorporation of six endodontic bacteria into an existing subgingival nine-species biofilm model. The counts of five out of nine strains in “basic biofilm” tend to decrease by the addition of some of the endodontic pathogens on HA discs. Only *C. rectus* counts increased by addition of *E. faecalis, S. aureus, P. nigrescens,* and *S. sputigena.* On dentin discs, *C. rectus* and *P. intermedia* counts increased by addition of the mentioned strains or *E. feacalis* alone, respectively. Based on this study, future investigations on endodontic infections can rely on this newly established endodontic-like multispecies biofilm model.

## Figures and Tables

**Table 1:** Species-specific primer sequences used in this study.

Organism	Sequence (5′ → 3′)	Reference
*Streptococcus anginosus*	fw: ACC AGG TCT TGA CAT CCC GAT GCT A rv: CCA TGC ACC ACC TGTC ACC GA	[31]
*Streptococcus oralis*	fw: ACC AGG TCT TGA CAT CCC TCT GAC C rv: ACCACCTGTCACCTCTGTCCCG	[31]
*Actinomyces oris*	fw: GCC TGT CCC TTT GTG GGT GGG rv: GCG GCT GCT GGC ACG TAG TT	[31]
*Veillonella dispar*	fw: CCC GGG CCT TGT ACA CAC CG rv: CCC ACC GGC TTT GGG CAC TT	[31]
*Fusobacterium nucleatum*	fw: CGC CCG TCA CAC CAC GAG A rv: ACA CCC TCG GAA CAT CCC TCC TTA C	[31]
*Campylobacter rectus*	fw: TCA CCG CCC GTC ACA CCA TG rv: CCG GTT TGG TAT TTG GGC TTC GAG T	[31]
*Prevotella intermedia*	fw: GCG TGC AGA TTG ACG GCC CTA T rv: GGC ACA CGT GCC CGC TTT ACT	[31]
*Porphyromonas gingivalis*	fw: GCG AGA GCC TGA ACC AGC CA rv: ACT CGT ATC GCC CGT TAT TCC CGT A	[31]
*Treponema denticola*	fw: TAA GGG ACA GCT TGC TCA CCC CTA rv: CAC CCA CGC GTT ACT CAC CAG TC	[31]
*Tannerella forsythia*	fw: CGA TGA TAC GCG AGG AAC CTT ACC C rv: CCG AAG GGA AGA AAG CTC TCA CTC T	[31]
*Enterococcus feacalis*	fw: CCG AGT GCT TGC ACT CAA TTG G rv: CTC TTA TGC CAT GCG GCA TAA AC	[34]

**Table 2:** Sequence and formamide concentrations for FISH Probes.

Probe ^1^	Target Organisms	Site ^3^	Formamide (%)	Sequence (5′–> 3′) ^2^	Reference
Efae470	*Enterococcus faecalis*	470–489	30	GAT ACC GTC AGG GGA CGT TC	[35]
FUS664	*Fusobacterium* spp.	664–683	40	CTT GTA GTT CCG CYT ACC TC	[36]
L-Pint649-2	*Prevotella intermedia*	649–667	40	CGT TGC GTG CAC TCA AGT C	[24]
Pint649	*Prevotella intermedia*	649–667	30–40	CGT TGC GTG CAC TCA AGT C	[37]
Pnig657	*Prevotella nigrescens*	657–675	40	TCC GCC TGC GCT GCG TGT A	[37]
Pmic740	*Parvimonas micra*	740–759	25	CTG AGC GTC AGT AAA AGT CC	[38]
Sspu439	*Selenomonas sputigena*	439–456	40	CGG TTT TCG TCC CGT GCA	This study
TrepG1-679	Treponemes Cluster 1, *(Treponema denticola* et rel.)	679–696	40	GAT TCC ACC CCT ACA CTT	[39]
Saur229	Staphylococcus aureus	229–246	40	CTA ATG CAG CGC GGA TCC	[40]

^1^ Probes were labeled at the 5′-end with FITC, Cy3, ROX, or Cy5, respectively. The designations of probes containing locked-nucleic-acid (LNA) substitutions start with L-. ^2^ Characters printed in bold indicate LNA substitutions. LNA incorporated DNA probes (LNA/DNA probes) have been described to improve significantly fluorescence intensity in comparison to conventional DNA probes with the same sequence [41]. ^3^ Targeted 16S rRNA region *E. coli* numbering [42].
